# Sesquiterpenoid lactones as potential anti-cancer agents: an update on molecular mechanisms and recent studies

**DOI:** 10.1186/s12935-022-02721-9

**Published:** 2022-10-07

**Authors:** Praveen Dhyani, Priyanka Sati, Eshita Sharma, Dharam Chand Attri, Amit Bahukhandi, Bekzat Tynybekov, Agnieszka Szopa, Javad Sharifi-Rad, Daniela Calina, Hafiz A. R. Suleria, William C. Cho

**Affiliations:** 1 Department of Biotechnology, Kumaun University, Bhimtal, 263 136 Uttarakhand India; 2 Graphic Era University, Dehradun, 248 001 Uttarakhand India; 3 Department of Molecular Biology and Biochemistry, Guru Nanak Dev University, Amritsar, 143 005 Punjab India; 4 High Altitude Plant Physiology Research Centre (HAPPRC), HNB Garhwal University, Srinagar Garhwal, 246 174 Uttarakhand India; 5 G.B. Pant National Institute of Himalayan Environment, Kosi-Katarmal, Almora, 263 643 Uttarakhand India; 6 Department of Biodiversity of Bioresources, Al-Farabi Kazakh National University, Almaty, Kazakhstan; 7 Department of Pharmaceutical Botany, Medical College, Jagiellonian University, Medyczna 9, 30-688 Kraków, Poland; 8 Facultad de Medicina, Universidad del Azuay, Cuenca, Ecuador; 9 Department of Clinical Pharmacy, University of Medicine and Pharmacy of Craiova, 200349 Craiova, Romania; 10 School of Agriculture and Food, Faculty of Veterinary and Agricultural Sciences, The University of Melbourne, Parkville, VIC 3010 Australia; 11 Department of Clinical Oncology, Queen Elizabeth Hospital, Kowloon, Hong Kong China

**Keywords:** Cancer, Sesquiterpenoid lactones, Anti-tumor, Apoptosis, Molecular mechanisms, Signaling pathway, Complementary medicine

## Abstract

Plants-based natural compounds are well-identified and recognized chemoprotective agents that can be used for primary and secondary cancer prevention, as they have proven efficacy and fewer side effects. In today's scenario, when cancer cases rapidly increase in developed and developing countries, the anti-cancerous plant-based compounds become highly imperative. Among others, the Asteraceae (Compositae) family's plants are rich in sesquiterpenoid lactones, a subclass of terpenoids with wide structural diversity, and offer unique anti-cancerous effects. These plants are utilized in folk medicine against numerous diseases worldwide. However, these plants are now a part of the modern medical system, with their sesquiterpenoid lactones researched extensively to find more effective and efficient cancer drug regimens. Given the evolving importance of sesquiterpenoid lactones for cancer research, this review comprehensively covers different domains in a spectrum of sesquiterpenoid lactones viz (i) Guaianolides (ii) Pseudoguaianolide (iii) Eudesmanolide (iv) Melampodinin A and (v) Germacrene, from important plants such as *Cynara scolymus* (globe artichoke), *Arnica montana* (wolf weeds), *Spilanthes acmella*, *Taraxacum officinale, Melampodium, Solidago* spp. The review, therefore, envisages being a helpful resource for the growth of plant-based anti-cancerous drug development.

## Introduction

Cancer affects 10 million people worldwide yearly, with cases anticipated to increase from 14.1 million to 21.6 million in 2012–2030 (https://www.who.int/news-room/fact-sheets/detail/cancer). Cancer can be effectively treated by reducing tumor weight and inhibiting cancer stem cells, with recurrence and treatment resistance [1–4]. Several medications for cancer have been developed in recent years due to attempts in previous decades to improve cancer treatment [5, 6]. These medications, however, have adverse effects, including drug-resistance development in patients over time and minimal efficacy in *in-vivo* systems due to low absorption [2, 7, 8]. As a result, finding novel cancer medicines that have a selective effect on cancer cells with proven efficacy, safety, fewer side effects, accessibility, and acceptance is a top priority for many pharmaceutical corporations and research groups [9–11]. To this, plant-based natural compounds have presented a promising and unique opportunity by offering chemo-preventive secondary metabolites, which are further well-identified and recognized active molecules for cancer prevention, both primary and secondary [12–15]. Sesquiterpenoid and sesquiterpenoid lactones (SLs) are various significant secondary metabolites in plants for humans and other species.

The Sesquiterpenoid lactones (SLs) elicit a variety of benefits, including neurodegeneration prevention, analgesic, antimigraine activity, and sedative actions; however, many researchers have extensively focused on its anti-tumor potential [16–18], natural compounds, and their synthetic derivatives such as Parthenolide from *Tanacetum parthenum*, Thapsigargin from *Thapsia,* Artemisinin from *Artemisia annua* L, currently being studied in clinical trials. In cancer studies, SLs have capabilities that allow them to treat the tumor and cancer stem cells while parting normal cells [19–21]. However, a quick, precise, and economical method for screening plants for anti-cancer potential is still necessary to discover effective tumor inhibitors for human usage [22].

Because of the above necessity and available options to mitigate cancer, this review article deals with different sesquiterpenoid compounds viz., (i) Guaianolides (ii) Pseudoguaianolide (iii) Eudesmanolide (iv) Melampodinin A, and (v) Germacrene, from essential plants such as *Cynara scolymus* (globe artichoke), *Arnica montana* (wolf weeds), *Spilanthes acmella*, *Taraxacum officinale, Melampodium, Solidago* spp. The review covers all aspects of knowledge about these compounds and plants, including their historical and contemporary medical applications, anti-tumor mechanisms, and related experimental pharmacological research. The study is a helpful element for developing anti-cancer treatments based on sesquiterpenoid lactones.

## Methodology

Bibliographic databases such as PubMed/Medline, Scopus, ScienceDirect, and Web of Science have been extensively analyzed to identify and compile relevant information on the anti-cancer molecular and cellular mechanisms of SLs. The following MeSH terms were used to search: “Antineoplastic Agents”, “Phytogenic/chemistry”, “Animals”, “Antineoplastic Agents”, “Phytogenic/pharmacology”, “Antineoplastic Agents”, “Apoptosis/drug effects,” “Cell Cycle/drug effects”, “Cell Line, Tumor”, “Phytogenic/therapeutic use”, “Lactones/pharmacology”, “Lactones/chemistry”, “Molecular Structure”, “Neoplasms/physiopathology”, “Neoplasms/drug therapy”, “Sesquiterpenes/chemistry”, “Sesquiterpenes/pharmacology”, “Sesquiterpenes/therapeutic use”, “Structure–Activity Relationship”, “Signal Transduction/drug effects”, “Plants, Medicinal”. The most relevant papers published on the anti-cancer properties of SLs in English have been included and cited. The papers were selected and retained based on the analysis of titles and abstracts, content on molecular mechanisms, and tested doses. In addition, relevant information on this topic has been taken from some official websites. Scientific plant names have been validated according to PlantList, and all chemical structures were described using PubChem and ChemSpider [23–25].

## Botanical sources of SLs

The SLs are most prevalent in the Asteraceae (Compositae) family, such as Artemisia, Helenium, and Arnica. This is among the most diverse and well-developed groups of higher plants (dicotyledonous angiosperms), 23,000 species and 1500 genera are subdivided into three subfamilies and seventeen tribes [26]. The Asteraceae family members are abundant in SLs and diterpenes and polyacetylenes secondary metabolites [27].

Farnesyl pyrophosphate (FPP) is used in plants to produce sesquiterpenoid lactones [28]. Plants' flowering heads and leaves are the most common sites where they can be found [29]. Lactifers are the most common source, but they can also be found in other cell types such as vacuoles and can make up to 3% of the total dry weight. In plants' Sesquiterpenoid lactones regulate plant growth and are necessary for allelopathic properties in plants. They are also important as herbivore repellants, antifungals, and antibacterials [30].

### Cynara scolymus

The perennial herb *Cynara scolymus* popularly referred to as globe artichoke, is in the Asteraceae family. Following the discovery of apigenin, luteolin, and cynarine, the plant has recently been necessary as a therapeutic herb [31]. Most of the cynarine in artichoke is concentrated in the substance of the foliage, while it can also be found in the dried leaves and stems [32].

Synonym and common name: *Cynara cardunculus* var. *scolymus*; Globe artichoke; French artichoke; green artichoke in the U.S [33].

Morphological description: *Cynara scolymus* is a 1.5 m (5 ft) × 1 m (3 ft 3 in) perennial plant that blooms from August to September and ripens from September to October. This species is hermaphroditic and is pollinated by honeybees, moths, and butterflies [34].

(i) Flower: The blossoms are purple and feature large heads of edible buds of 8–15-inch diameter and several triangular scales. The edible sections of the buds are the mushy basal sections of the involucral bracts and the base, also known as the heart. The choke, also known as the beard, is a cluster of immature florets in the bud's centre. Although, in older, more prominent blossoms, these are inedible.
(ii) Leaves: Bending, strongly lobed, shiny, pale leaves measure 50–83 cm in length.

Geographical distribution: *Cynara scolymus* is a native species of the Middle East, the Mediterranean basin in southern Europe, and North Africa [35].

Habitat: According to some historical, linguistic, and molecular documents, the domestication of artichokes (*Cynara scolymus*) from wild ancestors (*Cynara cardunculus*) may have occurred in Sicily in the first century. Ancient varieties are still cultivated in family gardens in the Midwestern part of Sicily (near Mazzarino) [35].

### Arnica montana

*Arnica montana* is a slightly noxious European annual herb. Noted for its big yellow flower head, it belongs to the *Asteraceae family*. Wolf weeds, leopard weeds, mountain tobacco, and mountain arnica are all names for the same plant*. A.* Montana is a rare plant that is recognized as an endangered species on the IUCN Red List and in several European nations' Red Data Books and Red Data Lists*.* [36]. Analgesic and anti-inflammatory properties are found in the medicinal herb *A. montana*. However, there isn't any evidence-based proof that these advantages or appropriate dosages exist. *Arnica montana* flower heads exclusively contain 0.2–0.8 percent pseudo guayanolid sesquiterpenes, though other major constituents are flavanone glycosides essential oils, sesquiterpene lactones, fatty acids, thymol, and pseudo guayanolid [37, 38].

Common name: wolf's bane, leopard's bane, mountain tobacco, and mountain arnica.

Morphological description: *Arnica montana* is a flowering plant, an aromatic and fragrant herbaceous perennial plant approximately 18–60 cm (7.1–23.6 inches) high. Its basic green, round-tipped oval leaves are bright in color and lie flat on the ground. In addition, they are slightly fluffy at the top, striped, and gathered in the rosette.

Geographical distribution: *Arnica montana* is distributed almost all over Europe. It does not exist in the British Isles, Italian, or Balkan Peninsula. It is also believed to be vanished in Lithuania and Hungary [39].

Habitat: *Arnica montana* flourishes in alpine pastures at nearly 3000 m (9800 ft), primarily in nutrient-depleted siliceous meadows or clay soils*.* It can also be found in undernourished marshes and wilderness in the highlands. However, Arnica does not grow on calcareous soils and is a highly reliable biomarker for nutrient-poor acidic soils [40]. Although it can be prolific in certain areas, it is rare in nature, primarily due to increased agricultural and commercial wildlife breeding practices.

### Spilanthes acmella

The Asteraceae *Spilanthes acmella* is a species that contains more than 60 species. The plant species *S. acmella* has been designated as endangered*.* [41]. This species includes a wide range of therapeutic and medicinal ingredients. The main components of this species, such as "Spilanthol" and "Acmellonate," are occasionally employed to relieve pain-related illnesses such as toothache and can stimulate salivation [42].

Synonym and common name: It is frequently referred to as the toothache remedy plant.

Morphological description: The flowers and leaves are intense and can be tingling or numb to the touch [43].

Geographical distribution: *S. acmella* is a tropical and subtropical plant widely dispersed worldwide, including the United States, Africa, Australia, Borneo, Malaysia, India, and Sri Lanka. It is an ornamental or medicinal plant cultivated all year in its native Brazil [44]. It is only found in India's south and central parts [45].

Habitat: *S. acmella* is a 40–60 cm tall perennial or short-lived plant found in marshes [43]. It has a weak vegetative proliferation rate or germination rate [46].

### Taraxacum officinale

*Taraxacum officinale*, dandelion, or common dandelion [47] is an Asteraceae family herbaceous perennial plant belonging to the Dandelion genus. Dandelions turn yellow flower heads into round balls, and many silver tufts of fruit are scattered in the wind. These balls are commonly referred to as "clocks" in British and American English. The name "blowball" is also used. Dandelions are medicinal and nutritious foods and drinks. The tender leaves can be cooked as a vegetable or eaten fresh in salads and sandwiches. The leaves are used for tea, the roots are used for coffee substitutes, and the flowers are used for wine and liquor.

Synonym and common name: Taraxacum is known by various names worldwide. In English-speaking countries, dandelions (from French dandelions to the leaves of the plant dandelions) are the most common names. It is also known as Wetabed, Dandelion, Fairy Clock, Priest Crown, Pig Snout, Dandelion, Milk Go One, Wild Endive, White Endive, Canker Weed, Puff Ball, Irish Daisy [48].

Morphological description: *Taraxacum officinale* grows from straight roots (usually unbranched) and produces multiple hollows, leafless flower stems: 470, usually 5–40 cm (2.0) high ~ 15.7 in.), but can be 70 cm (28 in.) high [49].

Leaves: Spatula-like leaves are deeply serrated, shiny, and placed in rosettes near the ground—the runcinate-pinnatifid or lobed oblanceolate foliage range from sparingly glandular to glabrous bottom sides. The leaves are 0.7–1.5 cm wide and 5–40 cm long and taper to a winged, petiolar base. The leaves' pronounced midrib varies in color from dark red-brown to pale yellow-green [50].

The formation of grooved leaf rosettes directs rainwater to the center, flowing into straight roots that are thick, dark brown, and practically black on the external.

Flowers: The yellow flowers, although they bloom all year, are climate and light-sensitive, opening at dawn and closing in the evening, opening in arid conditions and shutting in moist conditions. As the flowers mature, they close, and the petals wither, becoming puff balls with seeds scattered by the wind.

Root: The roots are cylindric, tapering, and branching slightly. It has a mild perfume and a pleasant sweetness to it. The inside of desiccated dandelions' roots is yellowish, spongy, and pithless. The robust and branching taproot can reach a circumference of 2–3 cm and a length of 1–2 m [50]. The lateral roots (arranged in two pairs) are spaced relatively evenly along their length. They wrap loosely around the root in a clockwise downward spiral.

Geographical distribution: Originally from Central Asia, this plant prefers a cooler climate and is spread almost worldwide. *Taraxacum* is a hardy plant that survives in damp, nitrogen-rich soils approximately 6000 feet above sea level. The majority of species are located in the Northern Hemisphere's temperate zones, with a concentration in northwest Europe [50].

Habitat: Dandelions grow on lawns, roadsides, turbulent banks, waterway banks, and other moist and nitrogen-rich soil areas in temperate regions of the world. Dandelion is most commonly seen as a weed, especially on lawns and roadsides [50].

### Melampodium spp.

Melampodium is a shrub genus and a member of the Asteraceae family. Melampodium is derived from the Greek terms (Melas), which implies "black," and (Podion), which means "foot." This relates to the color of the stems and roots [51]. Blackfoots are the popular name for members of this genus.

Synonym and common name: Butter daisy, Black foot.

Morphological description: This genus comprises annual, perennial, and shrub plants that reach a height of one meter. It tends to tumble over when fully matured.

Leaves: The leaves turn light green to a greyish-green color. On the other hand, the leaves are slender and roughly 2–5 cm long.

Flowers: The distal flower heads are around 2.5 cm in diameter. They produce a continuous display of white (most prominent in the three species of the white-rayed complex), cream, or yellow daisy-like ray florets, which a deeper orange disc floret center surrounds.

These functionally staminate disc florets are 8 to 10 broad. The five outer bracts are partially connected for around half of their length [52].

Fruit: Many fruits are like seeds (each consisting of fused inner varus bracts surrounding individual ray achenes) and have narrow scales at the apex.

Geographical distribution: These hardy plants thrive in tropical and subtropical environments such as Central America, the Southwest United States, California, Florida, the Caribbean, and South America.

Habitat: They like well-drained soils but can also thrive in rocky desert soils—their drought and heat resistance range from mild to severe.

### Solidago spp.

Solidago, also referred as goldenrod, is a flowering plant genus in the Asteraceae family that contains roughly 100 to 120 species [53]. Most of them are herbaceous perennials that grow in open regions like grasslands, prairies, and savannas.

Synonym and common name: Goldenrods.

Morphological description: Solidrod is a perennial plant that grows from woody ridges or rhizomes. Their stems range from 5 cm (2.0 in.) to over 1 m in height, from indigenous (creeping) to ascending or upright. Most species are unbranched, but some are branched at the top of the plant [54].

Leaves: The alternating leaves are about 46 "long and 1" wide, slightly smaller towards the top of the plant. They are almost linear in shape from the lanceolate and usually have small teeth along the edges [55]. If not, the edges are smooth. The stem has white hairlines, and the underside of the leaves is puberty [54].

Flowers: Some flower stalks emerge from the top of the plant in the form of spikes with tiny yellow flower clusters [56]. Each flower is less than 1/4 in. in diameter. The flowers appear at the top of each flower's stem and sometimes have a light scent. The flowering period is from summer to autumn, and one plant blooms for about 3 weeks. Achene has vertical ribs, trim hair, and small tufts that help disperse the wind.

Roots: The root system is fibrous, producing creeping rhizomes, stacking plants, and forming dense colonies.

Geographical distribution: Though some species flourish in Europe and Asia, they are native to North America.

Habitat: Solidago is one of the most critical autumn flowers from the eastern Great Plains to the Atlantic Ocean, and it may be spotted practically anywhere in woods, marshes, mountains, meadows, and roadside ditches [57].

## Ethnopharmacology

### Cynara scolymus

It is a herbaceous plant that originated in the Mediterranean and has since spread worldwide. Ancient Greeks, Romans, and Egyptians ate it as food and medicine, now a staple of the Dietary pattern. Typically, the inflorescence is fried and served as a meal. The leaflets have a strong flavor and are used to make aperitif liqueurs. They're even used to cure many conditions in traditional medicine, such as liver problems, jaundice, chronic albuminuria, dyspepsia, postoperative anemia, diuretic, and liver tonic (see Table 1) [58–61].

**Table 1:** Different plant parts used in the traditional medical system

Plant species	Plant parts	Preparation/extraction	Administration mode	Ailments cured	Country	Refs.
*Cynara scolymus*	Leaves	Leaf extract	Oral intake	Jaundice	French	[67]
				Embittering alcoholic, soft drinks, herbal tea	Europe	[61]
				Biliary tract, digestive action, scurvy, anemia	Tunisia	[60]
	Root bark	Dried plant parts are boiled with water	Oral intake	Febrifuge	Australia	[61]
*Arnica montana*	Fresh flowers	Decoction tincture and oil rubbing	Tinctures Ointments	Antirheumatic, hematomas, sprains	Italy	[62] [68] [69]
	Roots, flowers, and leaves			Bruises/sprains/rheumatic pain/skin inflammation/wounds	Spain	[63] [64]
*Spilanthes acmella*	Flowers and leaves	Dried	Oral intake	Toothache and throat problems	India	[70]
				Muscle pain, Headache, toothache	Bangladesh	[66]
	Whole plant			Cough	Haryana, India	[71]
				Head infections, itchiness	Jamalpur District, Bangladesh	[66]
	Flowers			Toothache	Tamil Nadu, India	[72]
	Whole plant			Anti-cancer agent	Indonesia	[72]
	Flower			Toothach, dysentery	Saurashtra region, Gujarat, India	[73]
	Leaves and flowers			Leucorrhoea, toothache, anti-inflammatory, astringent, gums, dysentery, antibacterial, anemia	Bogra District, Bangladesh	[73]
	Juice of inflorescence			Ulcer in mouth	Karnataka, India	[74]
	Flowers tincture			Sialagogue	Sri Lanka	[74]
	Cold infusion flowers			Diuretic, urinary calculi	Uva Province Sri Lanka	[74]
	Entire plant			Snakebite, rheumatic fever	Nigeria	[75]
	Leaves			Soup	Betsimisaraka Tanala people Madagascar	[75]
				Alcoholic hangover	Brazil	[76] [77]
*Taraxacum officinale*	Roots in combination with other herbs	Dried	Powder	The analgesic reduces the burning feeling of urination and regulates its outflow	India	[78]
	Leaves		Infusion	Ailments of the liver and bile ducts, viral and bacterial infections, cancer	Mexico	[79] [80]
	Leaves and roots	Crushed into a slurry and administer orally or topically		Liver problems, diuretic	Himalaya	[81]
	Leaves and root		Used in salads, dried, and fermented into wine	Blood and bowels are free of impurities	USA	[82]
	Flower and leaves		Infusion	Refreshing and digestible	Italy	[83]
	Whole plant		Powder	Hypertension	Ghana	[84]
	Whole plant		Decoction	Various skin inflammations, anti-haemorrhoids	Italy	[85]
	Leaves and roots		Decoction	Malaria	Venezuela	[86]
	Flowers		Cooking the blooms with sugar yields 'honey.'	Antitussive	Croatia	[85]
	Aerial part & Leaves		Infusion, Eaten as salad	Depurative, hepatic, renal discomfort, gastric ulcer	Bolivia	[87]
	Leaves and flowers		Decoction	Depurative, eupeptic. Gastronomic use	Italy	[88]
	Leaves and flowers		cooked or served in a salad	Anti-diabetic, diuretic, cholagogue, laxative	Italy	[83]
	Whole plant		Decoction	Liver diseases	Italy	[89]
	Leaves		Usage with salad	Depurative	Russia	[90]
	Leaves and roots		Infusion	Diuretic kidney stones, renal depurative	Peru Bolivia	[91]
	Leaves and roots		Dried leaves infusion or freshly prepared infusion The toasted root	Kidney stones, hepatodepurative, depurative, diuretic	Colombia	[91]
	Aerial part		Raw or fried	Food	Slovakia	[92]
	Flowers, leaves, roots		Raw, cooked, or boiled	Diuretic, respiratory suppressant, hypotensive, astringent to the intestine	Italy	[93]
	Leaves		As just a salad ingredient, or blanched or cooked as a vegetable	Food	Serbia	[94]
	Flower Leaves		Infusion	Infections of the stomach, urinary tract, menstrual cycle, lungs treatment	Kosovo	[95]
	Flower Leaf Root		Infusion. Milk-based decoction	Diabetes, rheumatism, anemia, menstrual irregularities, blood purification, corpus purification, biliary tract purification, digestion, loss of appetite, liver problem	Bosnia and Herzegovina	[96]
	Leaves Roots		Decoction Cataplasms	Diabetic, digestive, diuretic, and tonic. Constipation, liver, spleen, cardiac swelling, rheumatic aches are treated with root paste	Pakistan	
	The apical section of florets and the base leaves		As a snack or in salads, Roots that have been fried	Coffee alternative	Spain	[97]
	Leaves		The leaves are either fried in oil or simmered in water	Reduce the severity of hot flashes	Tibet	[98]
*Melampodium*	Leaves		Extract	Fever, malaria, flatulence, stomachache, colic, joint pain, muscle discomfort, palpitation, vertigo, rheumatism, jaundice, and anuria might occur Carminative and diuretic	Worldwide distribution	[99] [100]
	Leaves		Extract	Wound healing, antiulcer, antipyretic, anti-inflammatory, antipyretic		[101] [102]
*Solidago*	Whole plant		Fresh rhizomes	It's used to treat mouth and throat irritation, wounds and bleeding, urinary tract, nephritis, cystitis, and bladder malfunction, and it's also used in tea to help pass gallstones	Hungary	[103] [104]

### Arnica montana

It's been utilized in indigenous and homeopathic medical systems throughout most of North America and Europe for millennia. The decoction made from fresh leaves, flowers, and roots is widely used in the preparation of Tinctures and Ointments, which are utilized against many ailments like antirheumatic, hematomas, sprains, bruises, rheumatic pain, skin inflammation, and wounds [62–64].

### Spilanthes acmella

It is a Brazilian native commonly referred to “toothache plant” due to its conventional usage in treating dental discomfort. The species is one of the most frequent Amazonian herbal remedies used by the Amazon basin's lay inhabitants to treat tuberculosis. Traditionally, the flower juice of S*pilanthes acmella* is used to treat snakebite, dysentery, rheumatism, leucorrhea, mouth ulcers, and rheumatism in India and Bangladesh, while in Brazil, it is also used as an alcoholic hangover [65, 66].

### Taraxacum officinale

It is frequently recognized as a dandelion. It grows as a perennial plant throughout Europe, Asia, and North America. It's extensively used as a sedative in India to regulate urine discharge, minimize urine burning sensation, and treat diuretic and liver disorders [78, 81]. In Mexico, it is used to treat cancer, liverwort and bilious diseases, and microbial infections [79, 80]. In Italy, laxative, Depurative, Diuretic, eupeptic, stomachic, cholagogue, bitter-tonic, liver, and anti-diabetic illnesses are treated with it [83, 93]. It is also used to cure diabetes, swelling, and joint pains as a laxative for constipation and a diuretic tonic in Pakistan [105]. The predominant secondary metabolites are guaianolides and hydroxycinnamic acids, which are the main class represented by cynaropicrin and chlorogenic acid in leaves and play a vital role in biological activities, including hepatoprotective, antioxidant, antitumoral, antimicrobial, and anti-hyperlipidemic properties [106]. Eudesmanolide is the active principle compound of the plant, used to cure a variety of ailments like a diuretic [67], hepatoprotective, antiviral and anti-cancer, etc., through modern applications [107–110].

### Melampodium spp.

It is a sweet-smelling annual plant that thrives in the Caribbean, Africa, Colombia, Central America, and southern America. In Mexico, it is used to treat dysentery, fever, embolism, infections, discomfort, gastrointestinal issues, antiulcer, antipyretic, anti-inflammatory, and wound healing by using an infusion made from the floral portions of the plant. In Guatemala, the foliage relieves stomach aches, and the entire plant is used to cure influenza. Malaria, rheumatism, joint or muscular discomfort, vertigo, and stomachache are treated using plant leaves as diuretics [99–102]. The aerial parts of the plant Melampodium spp. contains Melampodinin A, used to treat the anti-nociceptive and anti-hyperalgesia [102].

### Solidago spp.

The plant's aerial portions have been employed in indigenous-style medicine for centuries as a spasmolytic, anti-inflammatory, and diuretic medication for various ailments, particularly as a urological medication for bladder irritation and kidney disorder, and cystitis [102, 103, 111, 112]. The whole part of the plant *Solidago spp.* contains germacrene, which is used through *in-vitro* and *in-vivo* methods for anti-inflammatory activity [103].

## General characterization and structure-anticancer activity relationship

**Fig. 1:**
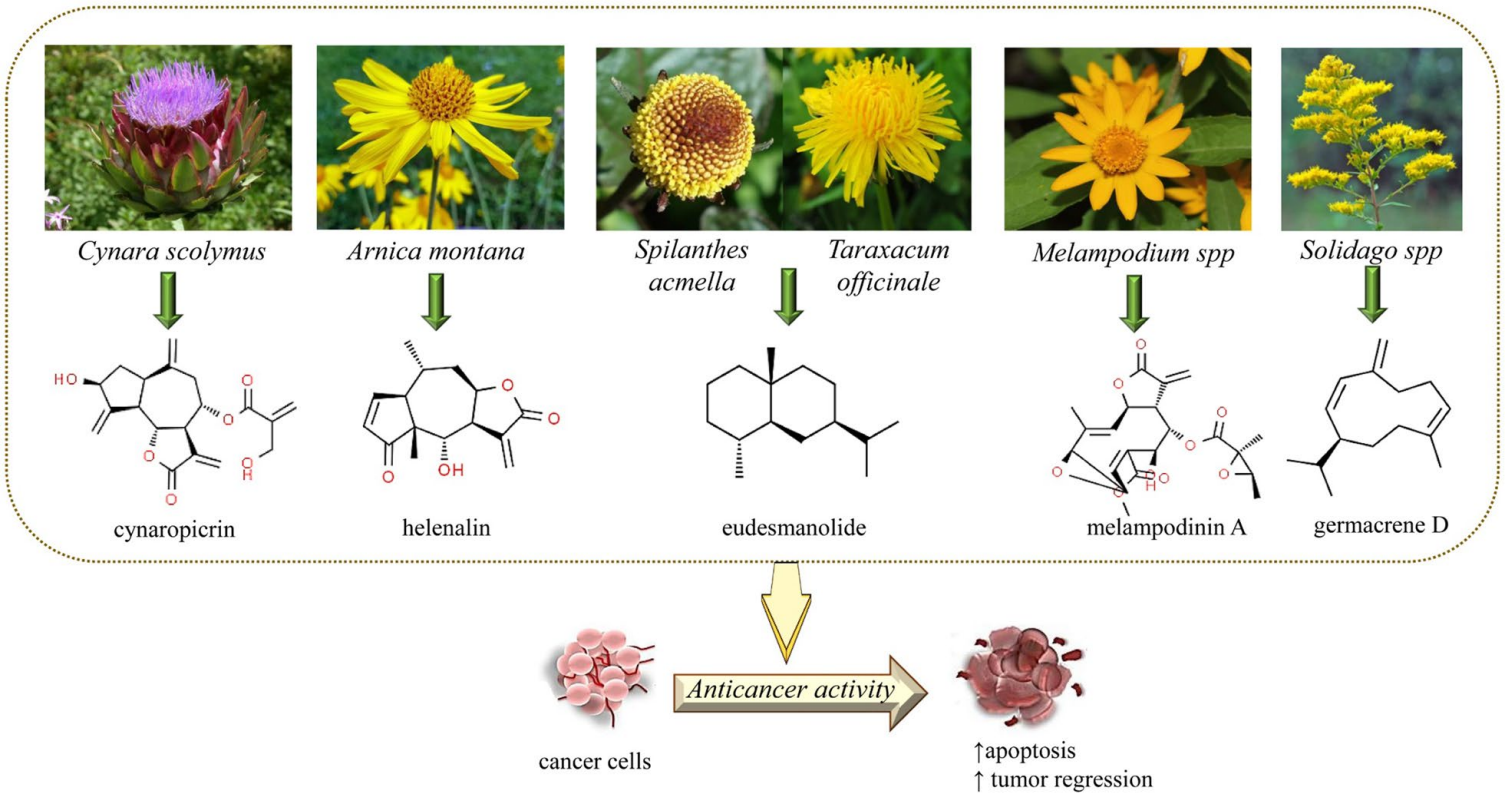
The most representative sources and chemical structures of SLs with anti-cancer properties

The SLs are natural substances with a 15-carbon framework that are mostly cyclic, could be hydrocarbon or oxygen-based, and can contain alcohol, ketone, or lactone group (Fig. 1). Within the Sesquiterpenoids superfamily, the SLs possess a lactone group and are bitter and colorless. In terms of structure, SLs are generally divided into five groups:

1. Germacranolides (ten-membered ring)
2. Pseudoguaianolides (5/7-bicyclic compounds)
3. Guaianolides (5/7-bicyclic compounds)
4. Elemanolides (6/6-bicyclic compounds)
5. Eudesmanolides (6/6-bicyclic compounds [113].

SLs have a wide variety of structural diversity, resulting in a wide range of biological activity. Three fundamental chemical properties which influence the biological actions of SLs are:

(i) Side chain and lipophilicity
(ii) Alkylating center reactivity
(iii) Molecule shape and electronic properties.

### Guaianolides

Guaianolides are sesquiterpene lactones made up of cyclopentane or cyclopentene and gamma-lactone. They have a cycloheptane or cycloheptene core that contains two structural isomers, 6,12-guaianolides and 8,12-guaianolides [114]. The IUPAC name for this compound is (3*S*, 3*aR*, 4*S*, 6*aR*, 9*aS*, 9*bR*)-4-hydroxy-3-methyl-6-methylidene-3*a*,4,5,6*a*,7,9,9*a*,9*b*-octahydro-3*H*-azuleno[4,5-b]furan-2,8-dione with molecular formula C_14_H_18_O_4_ and molecular weight is 250.29 g/mol (https://pubchem.ncbi.nlm.nih.gov/compound/15-Nor-guaianolide).

The biosynthetic origin or pathway for the majority of known guaianolides is unknown. Still, the route is thought to start with forming a germacrene lactone from farnesyl pyrophosphate [114]. It's made up of 3,6,9-trimethyldecahydroazuleno[4,5-b] furan-2(9bH)-one (1) or 3,5,8-trimethyldecahydroazuleno[6,5-b]furan-2(3H)-one (2), which belongs to a broad cluster of sesquiterpene lactones having chemotaxonomic and other biotic significances. Several more guaianolides, such as a-methylene guaianolides and guaianolides with an a,b-unsaturated carbonyl part, have been shown to have biological activity and used as chemotaxonomic markers [114].

Guaianolides exhibited diverse medicinal properties, such as high anti-tumor, anthelmintic, anti-schistosomal, antimicrobial, contraceptive, stimulating, antifeedant, root-growth, and germination-inhibiting actions [115]. These various biological activities make guaianolides valuable substances in pursuing new therapeutic leads [116]. In the series, the molecule guaianolide thapsigargin and its prodrugs have recently provided new insights into the drug discovery process. It further offers guaianolides an intriguing target because the biological source is commonly restricted, making it difficult to use as a long-term supply for getting the molecule on a kilo scale [114, 117].

In *Cynara scolymus,* guanolides comprise several compounds viz*.,* cynaropicrin, dehydrocynaropicrin, grosheimin, cynaratriol, 8-Epigrosheimin, aguerin A, aguerin B, 1b,13-Dihydrodesacylcynaropicrin-8-b-Dglucoside, 8-Deoxy-11,13-dihydroxy grosheimin, 8-Deoxy-11-hydroxy-13-chlorogrosheimin, 11-H-13-Methylsulfonylgrosheimin (grosulfeimin), 3b,8a,11b,13-Tetrahydroxy-10(14)-guaien-1a,4b,5a,6bH-6a,12-olide, cynarinin A, cynarinin B, cynarascoloside A, cynarascoloside BB and cynarascoloside C. These sesquiterpene lactone are primarily isolated from the roots, stalks, receptacles, aerial parts and leaves. These compounds are used against chronic inflammation, anti-osteoclastogenic and gastrointestinal evacuation [118–121].

### Pseudoguaianolide

Pseudoguaianolides are sesquiterpene lactones having a cis or trans-anellated lactone ring fused with seven five-membered rings, making them among the most prevalent natural compounds. Structurally Pseudoguaianolides contrast from guaianolides at the 5^th^ methyl position. It contained two groups, the less abundant helenanolides (helenalin, mexicanin, and aromatic) and the more abundant ambrosanolides group (ambrosin, parthenin, confertin, and damsin); in both, the methyl conformation in C-7 is β. Both the methyl configurations in C-7 include two groups, the less abundant helenanolides group (helenalin, mexicanin, and aromatic) and the more prominent ambrosanolides group (confertin, parthenin, damsin, and ambrosin).

The main synthetic procedures vary in how they build the hydroazulene skeleton, with one method relying on transannular cyclizations of a suitable cyclodecane or hydronaphthalene precursor. The culminating phases of the fusions, on the other hand, are the cautious building of the γ-butyrolactone and its α-methylenation [122]. The flower head of *Arnica montana* consists of pseudoguaianolide type sesquiterpene lactones e.g., helenalin, 11,13-dihydrohelenalin, and their esters, including 11,13-dihydrohelenalin acetate, helenalin acetate, 11,13-dihydrohelenalin isobutyrate, 11, 13-dihydrohelenalin a-methacrylate, 11, 13-dihydrohelenalin tiglinate, 11,13-dihydrohelenalin iso-valerianate, helenalin isobutyrate, helenalin a-methacrylate, helcnalin tignilate, helenalin iso-valerianate, helenalin 2-methyl butyrate, and tetrahydro helenalin isobutyrate, which are considered the primary active substances.

These compounds showed substantial anti-tumor and cytotoxic properties in anti-tumor agent screens. Helenalin seemed superior to 11, 13-dihydro helenalin in these effects [123]. Helenalin methacrylate, a new ester from helenalin, and helenalin acetate were isolated from the flowers of *Arnica montana*, and helenalin acetate is considered a contact allergen.

### Eudesmanolide

The IUPAC name for Eudesmanolide is 3aR,4aR,8aR,9aR)-4a-hydroxy-8a-methyl-3,5-dimethylidene-4,6,7,8,9,9a-hexahydro-3aH-benzo[f][1]benzofuran-2-one with molecular formula C_15_H_20_O_3_ and molecular weight is 248.32 g/mol (https://pubchem.ncbi.nlm.nih.gov/compound/Eudesmanolide-group). *Spilanthes acmella* isolates containing eudesmanolide II have a wide range of beneficial pharmacological actions, including antifungal, antipyretic, bioinsecticide, anticonvulsant, local anesthetic, anti-HIV, antimicrobial, aphrodisiac antioxidant, analgesic, a pancreatic lipase inhibitor, antinociception, diuretic, vasorelaxant [46, 124]. Recently, [125] reported that In terms of selectivity and activity, the aryl derivatives of eudesmanolide outperform known anti-cancer medicines such as etoposide.

### Melampodinin A

Melampodinin A has a considerable *in-vivo* repressive counteraction to lymphocytic leukemia (P-388) and is the main component of several *Melampodium* species. Melampodinin A's structure has been determined by ^1^H NMR correlations with melampodinin A acetate. The analysis reported the compound with a recognized absolute configuration [126]. The IUPAC name for the compound is methyl (1*R*, 2*E*, 4*S*, 6*R*, 7*E*, 9*S*, 10*S*, 11*S*)-9-acetyloxy-10-[(2*R*, 3*S*)-3-acetyloxy-2-hydroxy-2-methylbutanoyl]oxy-3-methyl-12-methylidene-13-oxo-5,1 dioxatricyclo[9.3.0.0]tetradeca-2,7-diene-8-carboxylate with molecular weight 522.5 g/mol and molecular formula is C_35_H_30_O_12_ (https://pubchem.ncbi.nlm.nih.gov/compound/Melampodinin).

### Germacrene

Germacrenes, or sesquiterpenes, are organic hydrocarbons and volatile. These are synthesized in many plant species for antibacterial and insecticidal qualities, but they also contribute as pheromones for insects. It has two important molecules, i.e., germacrene A and germacrene D (https://en.wikipedia.org/wiki/Germacrene). The IUPAC name for the compound is (1E,5E,8R)-1,5-dimethyl-8-prop-1-en-2-yl-cyclodeca-1,5-diene having molecular formula C_15_H_24_ with molecular weight 204.35 g/mol (https://pubchem.ncbi.nlm.nih.gov/compound/6436582).

The Isoprenoid biogenetic origin and synthesis, which is the building block of sesquiterpene germacrene D, has been extensively analyzed in *Solidago canadensis* through feeding experiments with 1-[5,5-d2]deoxy-d-xylulose-5-phosphate (D2-DOXP), [5-13C] mevalono lactone (13CMVL), and [1-13C]-d-glucose, predominantly via the methylerythritol phosphate pathway [127].

## Anti-carcinogenic activity of SLs: efficacy, signaling pathways and cellular mechanisms of action

### Guaianolides *(Cynara scolymus)*

Cynaropicrin, a guaianolide type sesquiterpene lactone, is a possible medication that inhibits leukocyte cancer cell invasion, migration, and metastasis [128]. Cynaropicrin is a probable drug for treating or preventing human cancers [129]. Cynaropicrin's anti-cancer action on U937 cells is caused by apoptosis activation by cell cycle stall in the G1/S stage. The therapeutic potential of cynaropicrin is diminished in the presence of N-acetyl-L-cysteine and L-cysteine, ROS scavengers, or rottlerin (a protein kinase (PK) C inhibitor). Cynaropicrin causes the proteolytic cleavage of PKC, PKC, and ROS to mediate the pro-apoptotic effect of the substance [129].

Effective suppression of STAT3 contributes to the reduction of anti-apoptotic genes, Bcl-2, and denocarc, in the DU145 cell line (human prostate cancer cell), which is ubiquitously expressed. In the DU145 cell line and THP-1 cells, cynaropicrin reduced STAT3 activation. Cynaropicrin activates S-glutathionylation of STAT3. It inhibits its phosphorylation via Michael's addition process, which produces a quick decrease in cellular GSH content dose-dependently. By causing STAT3 cysteine residues to undergo a redox-dependent post-translational change, cynaropicrin controls STAT3 functionality [130].

A new investigation on the anti-tumor action of cynaropicrin has revealed its delayed effect on skin photoaging promotes melanocyte and keratinocyte proliferation by inhibiting NF-κB transcription activity [131]. Recently, [132] showed that cynaropicrin in a dose-time-dependent fashion initiates apoptosis of Hela cells. Studies established that cynaropicrin interrupts the thioredoxin (Trx) system via thioredoxin reductase (TrxR) inhibition, leading to oxidation of Trx and accumulation of ROS in HeLa cells. Principally, cynaropicrin cytotoxicity is increased via the genetic knockdown of TrxR, depicting that the carcinogenic pharmacological outcome of cynaropicrin is associated with TrxR inhibition.

### Pseudoguaianolides (*Arnica montana*)

A sesquiterpene lactone from *A. Montana*, helenalin, mediates by impeding NF-Κb and p65 and anomalous stimulation of the NF-κB pathway. Helenalin causes sub-G1 halt, caspase cleavage, apoptosis, and a rise in autophagic marker concentrations. Helenalin reduced autophagic cell death by inhibiting LC3-B and Atg12 activation by suppressing caspase cleavage using the pan-caspase inhibitor Z-VAD-fmk, demonstrating that caspase functioning was required; for autophagic cell death. In a dose-time-dependent manner, helenalin decreased the expression of NF-B and p65. Exogenous p65 overexpression was linked with a cell death reduction; however, siRNA-mediated suppression increased autophagic cell death indicators and caspase cleavage, increasing cell death[133].

### Eudesmanolide (*Spilanthes acmella *and *Taraxacum officinale*)

*Spilanthes acmella* extract possesses anti-breast cancer activities that inhibit colony formation and metastasis in human t MCF-7 breast cancer cells [134]. Researchers have studied the anti-cancer effects of *S. acmella* extract on two types of cancer cell lines: colon (HT-29) and liver (HEP-2). HEP-2 and HT-29, with an anti-cancer activity of 771 ± 90 and 741 ± 03 percent, respectively, were effective. The possible mechanism proposed was a reduction in the ROS formation and induction of apoptosis involving growth regulators [135]. *S. acmella* can prevent cancer and DNA damage. *S. acmella* prevents and intercepts DNA damage by its activity against free radicals. Chinese hamster lung carcinoma (V79) and Dalton's lymphoma ascites (DLA), two cancer cell lines, were utilized to investigate several anti-cancer compounds. Only lymphoma cells were susceptible to the plant extract's anti-cancer properties; however, no significant inhibition was observed in carcinoma cells [136].

*Taraxacum officinale* extracts inhibit prostate and breast cancer cell invasion and growth. The methanol extract of Taraxacum officinale leaf repressed the invasion of LNCap prostate cancer cells and lowered the development of MCF-7AZ breast cancer cells [137]. An additional study demonstrated that *Taraxacum officinale* plant extract decreased breast cancer cell spread and growth by modulating the phosphatidylinositol 3-kinase (PI3K)/protein kinase B (AKT) pathway [138]. *Taraxacum officinale* causes cytotoxicity in human hepatic cancer cells, according to [139]. According to recent research, these plant extracts have been reported to cause programmed cell death in various cancer cells, including human leukemia, colorectal, prostate, and pancreatic cancer cells [140, 141]. Mechanism of action involves higher levels of ROS activating cellular stress machinery and sensitizing cancer cells to advance to apoptosis.

*In-vitro*, BCSCs formed microtumors, augmented the appearance of N-cadherin and Slug, reduced E-cadherin expression, and invaded the extracellular matrix (ECM). In two-dimensional (2D) and three-dimensional (3D) models of BCSC, the proliferation of BCSC was strongly reduced by dandelion extracts. The transcription of tumor necrosis factor-related apoptosis-inducing ligand (TRAIL) and TRAIL receptor 2 was enhanced in BCSCs treated with dandelion concentrates (TRAIL-R2; i.e., death receptor 5; DR5) [142]. Dandelion root extract (DRE) promotes cell death in human melanoma cells with high selectivity and efficacy while generating no harm in noncancerous cells. Human melanoma cell A375 exhibited characteristic apoptotic morphology after forty-eight hours, including nuclear condensation and phosphatidylserine shifting to the plasma membrane's outer leaflet. In A375 cells, DRE-induced apoptosis causes the commencement of caspase 8, representing the use of an extrinsic apoptotic mechanism to exterminate A375 cells [143]. The anti-tumor actions of *Taraxacum officinale* extract (aqueous-fermented) on a neonatal neuroblastoma cell line, SH-SY5Y, and Kelly revealed that Taraxacum causes apoptosis, mitochondrial integrity loss, inhibition of invasion and migration [144].

In mice with human prostate cancer xenografts, oral dosing of a mixture of Taraxacum officinale and lemongrass extract dramatically reduced tumor volume [140]. Taraxacum officinale dandelion extracts effectively reduced the development of breast cancer stem cells (BCSCs).

### Melampodinin A (*Melampodium spp.*)

The action mechanism of melampodinin A involves the seizure of the cell cycle’s G2/M stage, inhibiting cellular events vital for spindle formation resulting in abnormal mitotic spindle formation and function, thus, causing cell death [145]. The sesquiterpene lactones of *Melampodium* stimulate the apoptosis of DU145 cells and PC-3, arresting the GAP2/mitotic stage of dividing cells, and resulting in abnormal mitotic spindle formation [146]. In the SW1353 cell line (human bone chondrosarcoma cell line), sesquiterpene lactones impede tubulin polymerization and nuclear factor-B-mediated transcription, causing G2/M stall [145].

Melampodinin A was cytotoxic to DU 145 and PC-3 prostate cancer cells and HeLa cervical cancer cells. Melampodinin was cytotoxic to DU 145 prostate cancer cells, HeLa cervical cancer cells, and PC-3. Melampodinin A triggered a build-up of cells in the cell Melampodinin was found to be cytotoxic to PC-3, HeLa cervical cancer cells, and DU 145 prostate cancer cells cycle's at the G2/M phase, resulting in abnormal mitotic spindles, suggesting that cytotoxic effects of Melampodinin A involve mitotic spindle function inhibition and hence, also add to cell apoptosis [145].

### Germacrene (*Solidago spp.)*

*Solidago virgaurea* L. sesquiterpenes germacrene D (8.2–17.0%) is present in leaves and flowers. Compared to conventional medicine doxorubicin, a sesquiterpenes oil fraction of Solidago sp. grown in Egypt containing germacrene D exhibited promising cytotoxic efficacy against MCF-7, Hela, and Hepg2cell lines [147]. Anti-tumor activities (*in-vitro*) of germacrene were assessed in 3 distinctive cell lines, i.e., breast carcinoma cells (MCF7), cervix carcinoma cells (Hela), and human liver carcinoma cells (Hepg2). The autumn and winter oil samples contained more germacrene D as the significant component than the summer samples. All oil samples showed potent cytotoxic action to MCF-7, Hela, and Hepg2, cell lines, indicating its solid anti-tumor potential [147]. Anti-tumor activity was demonstrated in an essential oil extract of leaves of Solidago canadensis against Hela, SGC-7901, and SMMC-7721, with inhibitory doses of 68.1, 71.4, and 156.9 g/ml, respectively [148] [149].

**Fig. 2:**
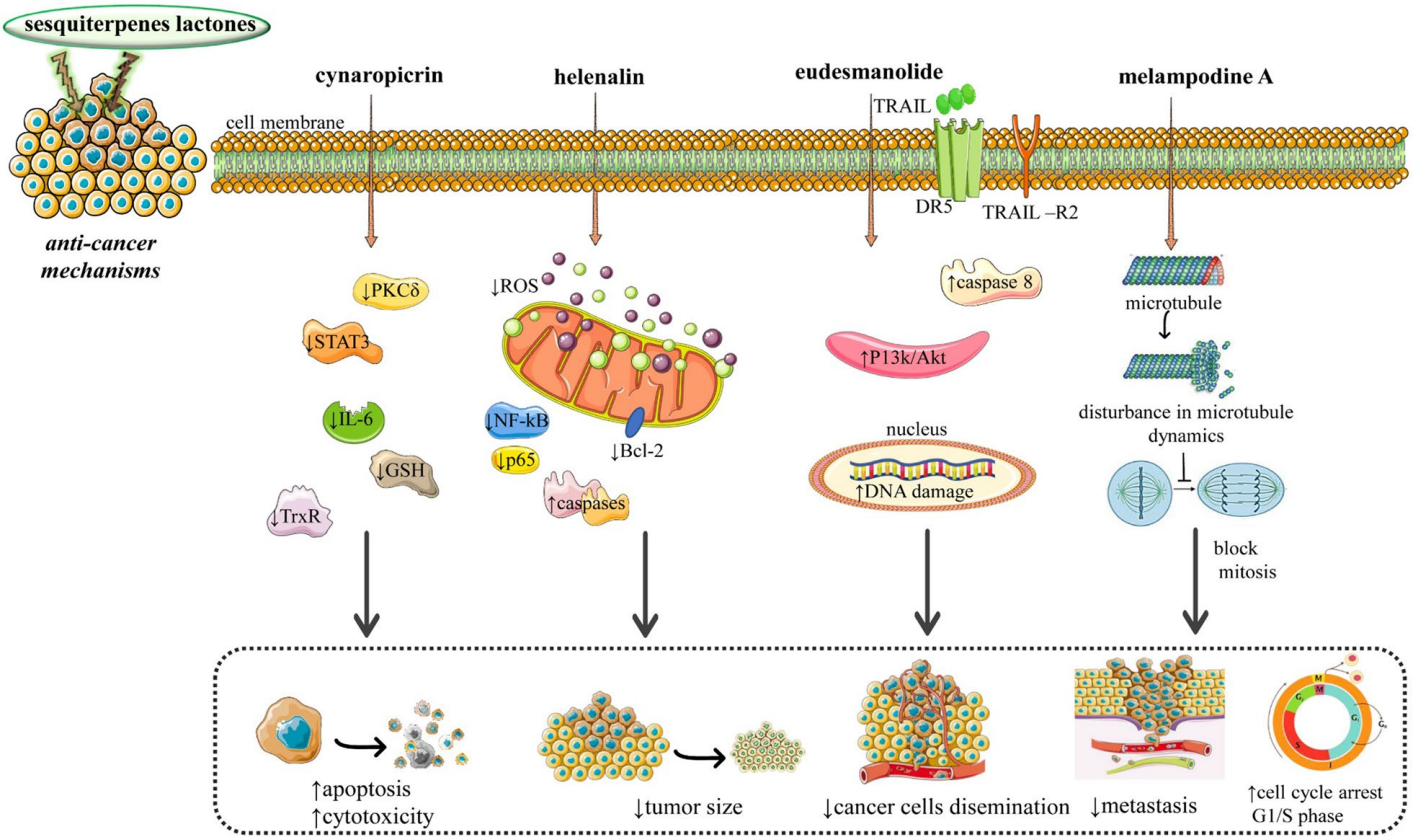
Diagram with the most important anti-cancer mechanisms and signaling pathways of SLs. Abbreviations and symbols: ↓decreased, PKCδ (Protein kinase Cδ), Signal transducer and activator of transcription 3 (STAT3), Interleukin 6 (IL-6), Glutathione (GSH), Thioredoxin Reductase (TrxR), Reactive oxygen species (ROS), nuclear factor kappa-light-chain-enhancer of activated B cells (NF-kB), B-cell lymphoma 2 (Bcl2), deoxyribonucleic acid (DNA)

The essential mechanisms are summarized in Fig. 2 and Table 2.

**Table 2:** Summarized experimental studies and anti-cancer mechanisms of action of SLs

Tested compound/source	Cancer tested model	Mechanisms of action	Refs.
Guaianolides *(Cynara scolymus) *Cynaropicrin	leukocyte cancer cells in vitro	↓invasion ↓migration ↓metastasis	[150]
	U937 cells in vitro	↑apoptosis ↑cell cycle stall at the G1/S stage	[151]
	DU145 human prostate cancer cells in vitro	↑anti-apoptotic genes ↑Bcl-2, ↓IL-6, ↓STAT3 ↓intracellular GSH	[152]
	Keratinocytes cells in vitro	↓TrxR ↓NF-κB	[132]
Pseudoguaianolides (*Arnica montana*) Helenalin	A2780 human ovarian cancer cells RKO colon carcinoma cancer cells MCF-7 breast adenocarcinoma cancer cells in vitro	↑autophagic cell death ↑caspase activity	[153]
Eudesmanolide (*Spilanthes acmella* and *Taraxacum officinale*)	MCF-7 breast cancer cells in vitro	↓ metastasis ↓colony formation	[154]
	HEP-2 HT-29 liver cancer cells in vitro	↓ROS ↑apoptosis ↓cancer cells growth	[155]
	DLA Dalton’s lymphoma ascites cells V79 Chinese hamster lung carcinoma in vitro	↑DNA damage ↓ROS	[156]
	MCF-7AZ breast cancer cells LNCap prostate cancer cells in vitro	↓cancer cells growth ↓PI3K/AKT	[157] [141]
	A375 human melanoma cells in vitro	↑apoptosis ↑caspase-8	[144]
	SH-SY5Y neuroblastoma cells in vitro	↑cytotoxic activity	
	mice in vivo	↓tumor size	[157]
Melampodinin A (*Melampodium spp.*)	PC-3 DU145 Hela in vitro	seizure of the cell cycle at the G2/M stage ↑abnormal mitotic spindle cycle ↑cytotoxicity	[145]
Germacrene (*Solidago spp.)* germacrene D	Hepg2 liver carcinoma cells breast MCF7 carcinoma cells cervix Hela carcinoma cells in vitro	↑cytotoxic activity	[147]
	SMMC- 7721 SGC-7901 Hela in vitro	↑cytotoxic activity	[158]

Symbols: ↑increase, ↓decrease

## Limitations and clinical gaps

SLs have demonstrated a practical and capable approach to chemoprevention and chemotherapy based on preclinical studies. However, SLs cannot be used as a first-line treatment for oncological conditions due to the following shortcomings:

i. Lack of clinical trials to confirm the efficacy and side effects, and toxicity of these compounds
ii. Lack of precisely characterized extracts and purified bioactive compounds. These extracts should be evaluated in more preclinical pharmacological studies and chemically characterized to determine the exact amounts of bioactive compounds that could be used in clinical trials.
iii. The quantities of bioactive compounds depend on the growth, cultivation, geographical area, and extraction methods used.
iv. Lack of nano pharmaceutical approaches to incorporate these SLs into nanocarriers in target organs to increase their bioavailability and effectiveness. Nanotechnologies in cancer aim to develop a new delivery system for bioactive compounds with the following goals:the supply of phytochemicals at a rate directly correlated with the body's needs, during chemotherapy and the transport of phytochemicals to the targeted tumors [158–160].
v. Insufficient in vivo studies and the lack of translational studies with the mentioned compounds to establish effective doses in humans.

Although SLs cannot be used as first-line in cancer treatment, they can be used as adjunctive therapy with cytostatic drugs. However, it is necessary to analyze the synergistic interactions between SLs with chemotherapeutic drugs. As a result, determining the optimal effective dose and the safety of SLs associated with chemotherapy remains the primary therapeutic challenge and limitations.

## Conclusion and future perspective

SLs such as Guaianolides, Pseudoguaianolides, Melampodinin A, Eudesmanolide, and Germacrene offer an excellent prospect for cancer prevention/cure as an attractive alternative cancer management method. Sesquiterpenoids restrict cellular progressions such as the cell cycle, mediate carcinoma cell inhibition through Programmed Cell Death, and cancer cell apoptosis through various biological functions. Plant-based medication research is progressing rapidly; thus, the quest for novel sources is ongoing. Plants including *Cynara scolymus, Arnica montana, Melampodium, Spilanthes acmella, Taraxacum officinale*, and *Solidago* spp. cultivated *in-vitro* and *in-vivo* can be used to extract sesquiterpenoid lactones. However, *in-vitro* culture is an encouraging instrument for increasing the concentration of the bioactive metabolite and is a valuable tool for conserving the concerned species, the *Spilanthes acmella*, *Taraxacum officinale*, and *melampodynin* spp. and *Solidago* spp still lacks a significant number of scientific studies concerning increasing (*in-vitro)* crucial Sesquiterpenoid lactones harvest. Therefore, a more comprehensive research program is warranted covering the domains of biotechnological studies based on the bio-resourcing of the sesquiterpenoid lactones from natural resources.

## Data Availability

Not applicable.
